# The effectiveness of caffeinated chewing gum in ameliorating cognitive functions affected by sleep deprivation

**DOI:** 10.5935/1984-0063.20220044

**Published:** 2022

**Authors:** AliReza Moradi, Farrokh Ghahremaninejad, Ehsan Hoseini, Mohammad Naseh Talebi, Soroush Lohrasbi, Sharareh Farahimanesh, Mohammad Nami, Habibollah Khazaei, Kamran Kazemi, Mohammad Mohammadi

**Affiliations:** 1 Kharazmi University, Department of Clinical Psychology, Faculty of Psychology & Educational Sciences - Tehran - Iran.; 2 Kharazmi University, Department of Plant Sciences, Faculty of Biological Sciences - Tehran - Iran.; 3 Institute for Cognitive Science Studies, Tehran - Iran.; 4 Shiraz University of Medical Sciences, Department of Neuroscience, School of Advanced Medical Sciences and Technologies - Shiraz - Fars - Iran.; 5 Instituto de Investigaciones Cientificas y Servicios de Alta Tecnologia (INDICASAT AIP), Neuroscience Center - Panama - Panama.; 6 Dana Brain Health Institute, Iranian Neuroscience Society-Fars Chapter - Shiraz - Fars - Iran.; 7 Senses Cultural Foundation, Academy of Health - Sacramento - California - United States.; 8 Institute for Cognitive Science Studies (ICSS), Department of Cognitive Neuroscience - Pardis - Tehran - Iran.; 9 Kermanshah University of Medical Sciences, Sleep Disorders Research Center - Kermanshah - Iran.; 10 Shiraz University of Technology, Department of Electrical and Electronics Engineering - Shiraz - Fars - Iran.

**Keywords:** CANTAB, Caffeine, Fatigue, Psychoactive Substances, Herbal Drinks

## Abstract

**Objectives:**

This investigation aimed to compare caffeinated gums with two different dosages of caffeine (200mg vs. 300mg) by assessing their effectiveness on the improvement of cognitive functions among Iranian individuals voluntarily suffering from 30 hours of sleep deprivation.

**Material and Methods:**

Thirty-four healthy male volunteers with ages from 28 to 35 years old were randomly assigned to either 200 or 300mg caffeine intake. Each participant completed CANTAB subtests to assess their core cognitive functions including MOT, RTI, RVP, and SWM before and after sleep deprivation, as well as after being treated with caffeinated gum.

**Results:**

The 300mg caffeine intake group indicated higher levels of enhancement of core cognitive functions compared with those in the 200mg caffeine intake group.

**Conclusion:**

This study suggests that the dose of 300mg of caffeine could effectively enhance the cognitive functions of Iranian individuals suffering from sleep deprivation.

## INTRODUCTION

Sleep is a biological requirement for energy conservation, recovery and maintenance of physiological systems as well as the brain plasticity, which is considered as an important factor for health^1,2^. Sleep deprivation is defined in terms of lack of sleep to provide sufficient alertness during a day. It has been suggested that long-term sleep deprivation has a deleterious impact on working memory, learning, and cognitive performance^3^. Furthermore, inadequate sleep has been associated with maladaptive behavioural and physiological functions^4,5^.

It has been suggested that sleep deprivation is linked with memory impairments and poor attentional skills as well as decreased activation in the dorsolateral prefrontal cortex (DLPFC)^6^. Moreover, sleep deprivation impedes learning performance and functional connections between DLPFC and hippocampus^7^. Studies examining total sleep deprivation demonstrated significant impairments of sustained attention, vigilance, cognitive processing speed as well as reaction times^8^. It is important to note that studies using more complex tasks showed that executive functions, such as working memory, risk assessment, divided attention and self-m onitoring are more vulnerable. These findings proposed that prefrontal areas of the brain are highly susceptible to sleep deprivation^9^.

Caffeine is a widely-used psychoactive and psychostimulant substance present in various herbal drinks, foods and over-thecounter drugs such as coffee, tea, cocoa, caffeinated chewing gum, energy drinks, cola beverages and some dietary supplements, usually in low to moderate doses without major side effects^10-12^. While about 60 plant species were found to contain caffeine, the most important cultivated resources of caffeine are *Coffea arabica* (Rubiaceae), *Camellia sinensis* (Theaceae), *Theobroma cacao* (Malvaceae), *Cola nitida* (Malvaceae) and *Paulliania cupana* (Sapindaceae)^13,14^.

Many individuals consume caffeine to enhance their impaired performance affected by sleep deprivation^15,16^. The structure of caffeine is similar to that of neuromodulator adenosine, which is formed by ATP synthesis/breakdown^11,17^. There are four G-protein-coupled adenosine receptors including A1, A2a, A2b and A3^18^, each of which has its own distinctive pharmacological and tissue distribution profile^19^. The density and sensitivity of adenosine receptors might be different among individuals, however, as the caffeine intake of an individual increases, the adenosine receptors are up-regulated^15^.

Average daily caffeine intake per capita show a considerable variation in different countries, the example of which are as follows (mean ± SD): 31.3mg in Greece^20^, 36.5mg in Romania^20^, 66.8mg in Spain^20^, 67.8mg in South Korea^21^, 86±4.74mg in China^22^, 103mg in Hungary^20^, 130mg in Brazil^23^, 138.2mg in the United Kingdom^20^, 139.3mg in Italy^20^, 154.5mg in France^20^, 164.9mg in Australia^24^, 191±129mg in Switzerland^25^, 193mg in the United States^26^, 205.5mg in Sweden^20^, 236mg in Finland^20^, 238mg in Germany^20^, 258.5mg in Netherlands^20^, and 319.4mg in Denmark^20^.

Consequently, caffeine is found to be effective to enhance impaired performance related to sleep deprivation^16,27^. The effectiveness of caffeine depends on various factors, including the dosage, the nature of the performing task and the degree of sleep deprivation, as well as genetic factors and habituation^15,28,29^.

While caffeine could be effectively delivered to the consumers by various means, chewing gum showed to be a promising tool in various studies^30-33^. While the total caffeine absorption is more or less the same in various delivery methods, the caffeine content delivered by chewing gum via buccal mucosa was shown to be absorbed faster than that in methods such as capsule delivery and absorption in gut^33^.

Considering that sleep deprivation and sleep restriction have negative impacts on motor function, cognitive performance and mood^34^, in addition to the fact that caffeine has an effect on impaired performance^27^, the principal aim of the present study was to examine the effectiveness of 200mg and 300mg doses of caffeinated chewing gum in ameliorating cognitive performances, such as reaction time, vigilance, attention and working memory induced by sleep deprivation in the Iranian individuals by means of Cambridge Neuropsychological Test Automated Battery (CANTAB), a simple and computerized test without linguistic or cultural bias^35^.

## MATERIAL AND METHODS

### Participants

Thirty-four male volunteers *(Mage*=34.7, *SD=5.8)* were randomly assigned in two groups (the 200mg caffeine intake and the 300mg caffeine intake). The two groups were matched in terms of age and levels of education. All participants had a bachelor’s and master’s degrees and were chosen from different communities who work full-time night shifts, evening shifts and rotational shifts. Moreover, all subjects were healthy considering physical and psychological aspects. Furthermore, participants were interviewed in order to find out if they have previous cognitive difficulties. Participants were eligible if they had no history of psychotic and physical disorder, alcohol or other drug dependence, smoking habit and learning disabilities.

### Materials

**Caffeinated chewing gum:** non-commercial caffeinated chewing gums containing 200mg and 300mg of caffeine were ordered to be produced by the MasterFoodeh Company, Tehran, Iran, following the method standardized by Aslani and Jalilian (2013)^36^.

**Cambridge Neuropsychological Test Automated Battery (CANTAB):** is well-known for its high sensitiveness to positive and negative pharmacological, genetic and environmental effects in the individuals studied^37^. This test is used to evaluate particular cognitive features of the brain, especially those connected with medial temporal and frontal areas; CANTAB Motor Screening Task (MOT), to measure reaction time and evaluate the psychomotor function to be intact to perform more advanced cognitive testing; CANTAB Reaction Time (RTI), to evaluate response speed and estimate vigilance; CANTAB Rapid Visual Information Processing (RVP), to evaluate complex features of attention; and CANTAB Spatial Working Memory (SWM), to provide information about working memory^38^.

### Procedure

This research was carried out with the approval of the review board and ethics committee of the BLINDED. Ethical approval was obtained from Institutional Review Board of Kharazmi University, Tehran, Iran (IR.KHU.REC.1398.013). After obtaining informed consent, participants were assessed at three time points: baseline, after 30 hours of sleep deprivation and 30 minutes after the consumption of caffeine chewing gum. Following baseline assessments, participants were randomly allocated to either the 200mg caffeine intake *(*n*=*19) or 300mg caffeine intake (n*=*15). Each assessment included an interview and the completion of four CANTAB subtests, core cognitive functions, such as motor screening task (MOT), reaction time (RTI), rapid visual information processing (RVP), and spatial working memory (SWM). All the assessments were administered by a trainer blind to hypotheses and group allocation. In order to carry out the study, first, the participants were in a controlled condition for twenty-four hours (from 6 a.m. on the first day to 6 a.m. on the second day) in which they had enough sleep and normal servings of food, and they were careful not to take any caffeine, tea or coffee for instance. Then they underwent sleep deprivation for thirty hours (from 6 a.m. on the second day to 11:59 a.m. on the third day) while they had physical and mental activities. Moreover, they were careful not to take a nap or take any caffeine, such as tea or coffee. In addition, they had physical activity in their free time. Next, they took the first set of the CANTAB tests, and after that, they received 200mg and 300mg caffeine chewing gum. Half an hour later, they took the second set of the CANTAB tests.

### Data analysis plan

We conducted a series of 2 (the 200mg caffeine intake vs. the 300mg caffeine intake) x 3 (baseline, after 30 hours, after 30 minutes) mixed analysis of variance (ANOVAs) for each CANTAB subtests including MOT, RTI, RVP, and SWM. Eta squared effect size were evaluated and reported for ANOVA analyses, with 0.01 considered as small, 0.06 as medium, and 0.14 considered as large. Post-hoc*/*-tests and Cohen *d* effect sizes were calculated for significant interactions.

## RESULTS

Baseline means and standard deviation for four CANTAB subtests for the three time points are presented in Table 1. The two groups did not significantly differ in terms of age, marital status, education and the baseline of four CANTAB subtests. All participants completed all three assessment points.

**Table 1:** Descriptive statistics for 200mg and 300mg caffeine intake groups.

	200 mg		300 mg	
	Mean	Std. Deviation	Mean	Std. Deviation
Age - Years	35.2	6.7	33.6	4.8
MOT - Baseline	846.05	199.76	769.57	158.12
MOT - Sleep deprivation	881.28	235.08	756.11	129.87
MOT - Caffeine intake	754.33	171.63	670.47	103.62
RTI - Baseline	370.83	146.97	307.84	45.28
RTI - Sleep deprivation	375.49	123.30	327.23	55.31
RTI - Caffeine intake	342.54	50.25	312.79	33.87
RVP (A-Prime) - Baseline	0.910	0.041	0.92	0.044
RVP (A-Prime) - Sleep deprivation	0.920	0.045	0.94	0.044
RVP (A-Prime) - Caffeine intake	0.950	0.036	0.96	0.037
RVP (Response latency) - Baseline	470.61	121.75	505.32	121.36
RVP (Response latency) - Sleep deprivation	470.88	97.86	487.98	88.51
RVP (Response latency) - Caffeine intake	434.68	80.07	465.72	68.44
SWM (Between errors) - Baseline	12.78	12.59	19.66	16.17
SWM (Between errors) - Sleep deprivation	19.36	20.49	20.46	20.52
SWM (Between errors) - Caffeine intake	18.84	17.58	14.00	15.34
SWM (Strategy) - Baseline	29.63	5.12	32.26	6.25
SWM (Strategy) - Sleep deprivation	29.36	6.39	32.46	6.12
SWM (Strategy) - Caffeine intake	30.52	5.82	29.80	6.75

Abbreviations: MOT = Motor screening task; RTI = Reaction time; RVP = Rapid visual information processing; SWM = Spatial working memory; the exact p-values were indicated in the manuscript.

### CANTAB motor screening task (MOT)

**Figure 1:**
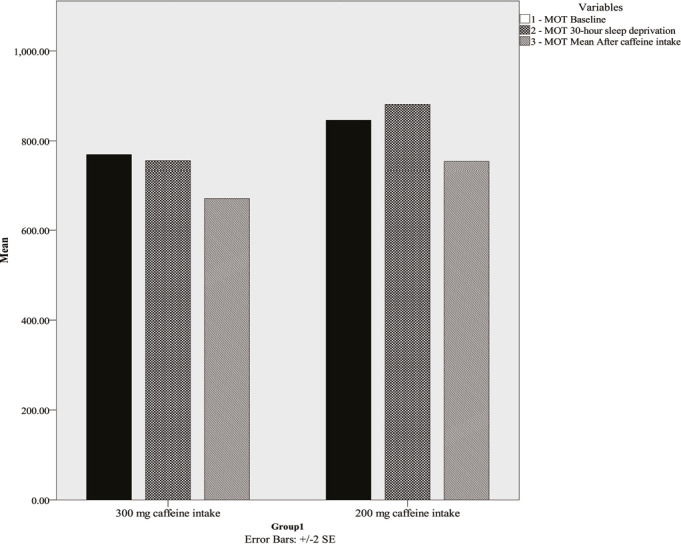
Motor screening task for the 200mg caffeine intake and 300mg caffeine intake groups at baseline, after 30 hours sleep deprivation and 30 minutes after caffeine consumption, values are mean ± SE.

Group means for MOT for the three time points are presented in Figure 1. A 2 (the 200mg caffeine intake vs. the 300mg caffeine intake) x 3 (baseline, after 30 hours, after 30 minutes) mixed ANOVA was conducted. The time main effect, F(2,64)=5.88, *p*=0.00, n^*2*^=0.15, and group main effect, F(1,32)=4.20, *p*=0.049, n^2^=0.11, were significant while interaction, F(2,64)=0.29, *p*>0.05, n^2^=.009, was not significant. Further post-hoc t-test analyses showed that while the two groups did not differ at baseline, the 300mg caffeine intake group showed significantly improvement on MOT task after 30 minutes, *t*(14)=3.21, *p*<0.001, than that in the 200mg caffeine intake group.

### CANTAB reaction time (RTI)

**Figure 2:**
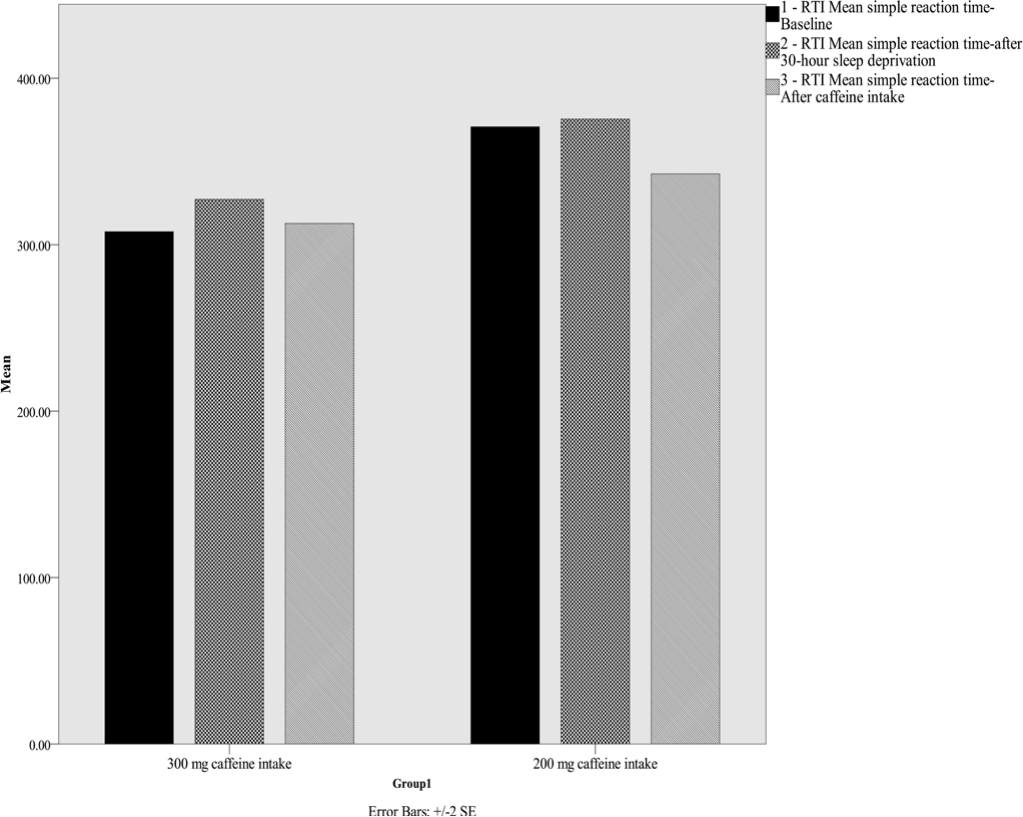
Reaction time task for the 200mg caffeine intake and 300mg caffeine intake groups at baseline, after 30 hours sleep deprivation and 30 minutes after caffeine consumption, values are mean ± SE.

Group means for RTI for the three time points are presented in Figure 2. A 2 (the 200mg caffeine intake vs. the 300mg caffeine intake) x 3 (baseline, after 30 hours, after 30 minutes) mixed ANOVA was conducted for simple choice reaction time. The time main effect, F(2,64)=5.38, *p*=0.02, n^2^=0.14, and the group main effect, F(1,32)=4.25, *p*=0.04, n^2^=0.11, were significant, while interaction, F(1,32)=0.34, *p*>0.05, n^2^=0.01, was not significant. Further post-hoc t-test analyses showed that the two groups did not significantly differ after 30 minutes.

### CANTAB rapid visual information processing (RVP)

**Figure 3:**
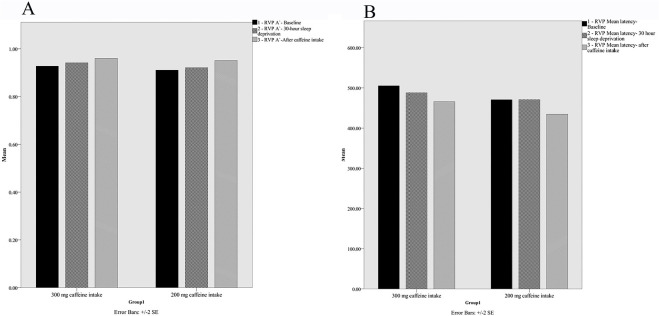
Rapid visual information processing task, A-Prime (**A**) and mean response latency aspects (**B**), for the 200mg caffeine intake and 300mg caffeine intake groups at Baseline, after 30 hours sleep deprivation and 30 minutes after caffeine consumption, values are mean ± SE.

Group means for RVP for the three time points are presented in Figure 3. A 2 (the 200mg caffeine intake vs. the 300mg caffeine intake) x 3 (baseline, after 30 hours, after 30 minutes) mixed ANOVA was conducted for two core features: A-Prime and mean response latency. In the case of A-Prime, the time main effect, F(2,64)=15.41, *p*=0.00, *p^2^*=0.32, was significant, while the group main effect, F(1,32)=1.58, *p*>0.05, n^2^=0.04, and the interaction, F(2,64)=0.36, *p*>0.05, n^2^=0.01, were not significant. In the case of mean response latency, the time main effect, F(2,64)=2.30, *p*>0.05, n^2^=0.06, and the group main effect, F(1,32)=1.07, *p*>0.05, n^2^=0.03, were not significant, while the interaction, F(1,32)=0.83, *p*=0.00, n^2^=0.20, was significant. Further post-hoc t-test analysis indicated that the two groups did not significantly differ after 30 minutes on A-Prime and mean response latency. However, the 200mg caffeine intake showed significant difference on A-prime core outcome after 30 minutes, *t*(18)=-3.47, *p*=0.003.

### CANTAB spatial working memory (SWM)

**Figure 4:**
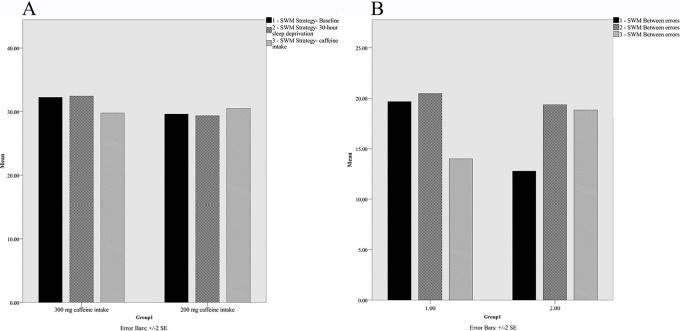
Spatial Working Memory task, strategy aspects (A) and between errors (B), for the 200 mg caffeine intake and 300 mg caffeine intake groups at Baseline, after 30 hours sleep deprivation and 30 minutes after caffeine consumption, values are mean ± SE.

Group means for SWM for the three time points are presented in Figure 4. A 2 (the 200mg caffeine intake vs. the 300mg caffeine intake) x 3 (baseline, after 30 hours, after 30 minutes) mixed ANOVA was conducted for two core features: between errors and strategy. In the case of between error feature, the time main effect, F(2,64)=1.34, *p*>0.05, n^2^=0.04, the group main effect, F(1,32)=0.04, *p*>0.05, n^2^=0.001, and the interaction, F(2,64)=2.61, *p*>0.05, n^2^=0.07, were not significant. Further post-hoc t-test analyses showed that the two groups did not significantly differ after 30 minutes. In the case of strategy core feature, the time main effect, F(2,64)=0.73, *p*>0.05, n^2^=0.02, the group main effect, F(1,32)=0.04, *p*>0.05, n^2^=0.001, and the interaction, F(2,64)=0.75, *p*>0.05, n^2^=0.02, were not significant.

Further t-test analyses showed that the 300mg intake group showed significantly improvement on strategy feature of SWM task after 30 minutes, *t*(14)=2.34, *p*=0.03, than that in the 200mg caffeine intake group.

It can be concluded that only in the 300mg caffeine intake group the MOT and SWM (the strategy aspect) were significantly differed after 30 minutes of caffeine chewing gum consumption. However, in the 200mg caffeine intake group A-prime core outcome of RVP subtest was significantly differed after 30 minutes of caffeine chewing gum consumption.

## DISCUSSION

Considering the fact that genetic factors and the degree of habituation could considerably alter the effectiveness of caffeine, to the best of our knowledge, this is the first study determining the effective dose of caffeine for ameliorating cognitive functions affected by sleep deprivation among the Iranian population.

Tea, carbonated drinks and coffee are the most notable sources of caffeine intake in Iran. Iranian people are generally preferring to drink black tea rather than coffee, therefore, consuming smaller amounts of caffeine per serving/portion^39^. The average daily intake of caffeine in the Iranian population is yet to be systematically determined in a nation-wide study; however, combining various sources would help to get a fairly accurate estimation. The average daily consumption of tea was estimated to be around 1,243ml per capita^40^, and the caffeine content of various brands of tea in Iran were estimated to range from 12.3518.75mg/l^41^. However, Balentine et al. (2019)^42^, had estimated the average consumption of tea in the Iranian population to be around 1.65 cup per day, each cup containing 30mg of caffeine. Therefore, daily caffeine intake from tea would be ranged from 15.35-49.5mg per capita. In addition, the average daily intake of caffeine from carbonated drinks and coffee in the Iranian population were estimated to be 1,253mg per capita (0,0179mg/kg body weight) and 0,00084mg per capita (0,000012mg/kg body weight), respectively^43^.

In conclusion, the average daily intake of caffeine in the Iranian population could be estimated to range from 16,60384 to 50,7538mg per capita, well below than that in many European and North American Countries and within a similar range of that in East Asian Countries with a prevalent tea drinking culture.

The aim of this study was to examine the effectiveness of 200mg and 300mg doses of caffeine on enhancing cognitive performance of Iranian individuals suffering from sleep deprivation. The findings of the study indicated that after thirty hours of sleep deprivation, participants’ functions in subtests of CANTAB including MOT, RTI, RVP and SWM decreased significantly. According to studies, sleep deprivation decreases the levels of memory, attention and executive functions^44,45^. Our findings were in line with those of another research indicating that sleeplessness has an impact on executive functions^46^. Our findings were also consistent with other studies regarding the effects of sleep deprivation on reaction time^47-49^.

The findings of the present study indicate the efficacy of 300mg caffeine intake in the enhancement of the MOT and SWM (the strategy aspect) subtests of CANTAB after 30 minutes of caffeine chewing gum consumption, while the 200mg caffeine intake was not confirmed to be effective. Based on studies, the consumption of 200mg of caffeine restore performance to near baseline levels after 24 hours of sleep deprivation^50^. However, after a 24-hour period of sleeplessness, a higher dosage of caffeine is needed^51^. It can be assumed that, based on the caffeine mechanism of action, the 300mg caffeine chewing gum improved cognitive performance. Similarly, other studies showed that the administration of 300mg caffeine decreased simple reaction time in addition to increasing alertness^52^. Furthermore, caffeine intake is effective in improving mental functions and enhancing cognitive functions after sleep deprivation^53^.

It is noteworthy that the ability to process and respond to information received from the environment plays a key role in effective functioning, especially in situations where rapid decision-making and fast reaction time are needed. Sleep deprivation has a significant impact on psychomotor speed, which includes both mental processing and physical movement. The slower the psychomotor speed, the more decrease in cognitive functioning might be observed. As a result, the findings of the present study could be beneficial for jobs, which require being awake, such as night shift drivers and nurses, and they could suggest an effective dosage of caffeine to enhance cognitive functions and restore alertness after a period of sleeplessness.

As for the limitations of this study, it could be pointed out that the sample size was rather small and consisted of only male participants, who were at a certain age range. In addition, other doses of caffeine could have been tested. Moreover, the participants could have been recruited from other parts of the country as well. Finally, this study provides a basis for future research using substances other than caffeine.

## References

[1] Roth TC, Rattenborg NC, Pravosudov VV (2010). The ecological relevance of sleep: the trade-off between sleep, memory and energy conservation. Phil Trans Royal Soc Biol Sci.

[2] Ohayon M, Wickwire EM, Hirshkowitz M, Albert SM, Avidan A, Daly FJ (2017). National Sleep Foundation’s sleep quality recommendations: first report. Sleep Health.

[3] Kaliyaperumal D, Elango Y, Alagesan M, Santhanakrishanan I (2017). Effects of sleep deprivation on the cognitive performance of nurses working in shift. J Clin Diagn Res.

[4] Alhola P, Polo-Kantola P (2007). Sleep deprivation: impact on cognitive performance. Neuropsychiatr Dis Treat.

[5] Palagini L, Bastien CH, Marazziti D, Ellis JG, Riemann D (2019). The key role of insomnia and sleep loss in the dysregulation of multiple systems involved in mood disorders: a proposed model. J Sleep Res.

[6] Yoo SS, Hu PT, Gujar N, Jolesz FA, Walker MP (2007). A deficit in the ability to form new human memories without sleep. Nat Neurosci.

[7] Chee MWL, Chuah YML (2007). Functional neuroimaging and behavioral correlates of capacity decline in visual short-term memory after sleep deprivation. Proc Natl Acad Sci.

[8] Thomas M, Sing H, Belenky G, Holcomb H, Mayberg H, Dannals R (2000). Neural basis of alertness and cognitive performance impairments during sleepiness. I. Effects of 24 h of sleep deprivation on waking human regional brain activity. J Sleep Res.

[9] Taber KH, Hurley RA (2006). Functional neuroanatomy of sleep and sleep deprivation. J Neuropsychiatry Clin Neurosci.

[10] Cappelletti S, Daria P, Sani G, Aromatario M (2015). Caffeine: cognitive and physical performance enhancer or psychoactive drug?. Curr Neuropharmacol.

[11] Carvey CE, Thompson LA, Mahoney CR, Wesensten NJ (2012). Sleep Deprivation, Stimulant Medications, and Cognition.

[12] Temple JL, Bernard C, Lipshultz SE, Czachor JD, Westphal JA, Mestre MA (2017). The safety of ingested caffeine: a comprehensive review. Front Psychiatry.

[13] Arnaud MJ (1987). The pharmacology of caffeine. Prog Drug Res.

[14] Ashihara H, Crozier A (2001). Caffeine: a well known but little mentioned compound in plant science. Trends Plant Sci.

[15] McLellan TM, Caldwell JA, Lieberman HR (2016). A review of caffeine’s effects on cognitive, physical and occupational performance. Neurosc Biobehav Rev.

[16] Irwin C, Khalesi S, Desbrow B, McCartney D (2020). Effects of acute caffeine consumption following sleep loss on cognitive, physical, occupational and driving performance: a systematic review and meta-analysis. Neurosc Biobehav Rev.

[17] Barsotti C, Ipata PL (2004). Metabolic regulation of ATP breakdown and of adenosine production in rat brain extracts. Int J Biochem Cell Biol.

[18] Fredholm BB (1995). Adenosine, adenosine receptors and the actions of caffeine. Pharmacol Toxicol.

[19] Landolt HP (2008). Sleep homeostasis: a role for adenosine in humans?. Biochem Pharmacol.

[20] European Food Safety Authority (EFSA) (2010). Scientific opinion on African swine fever. EFSA J.

[21] Lim HS, Hwang JY, Choi JC, Kim M (2015). Assessment of caffeine intake in the Korean population. Food Addit Contam Part A Chem Anal Control Expo Risk Asses.

[22] Shao XT, Cong ZX, Liu SY, Wang Z, Zheng XY, Wang DG (2021). Spatial analysis of metformin use compared with nicotine and caffeine consumption through wastewater-based epidemiology in China. Ecotoxicol Environ Safety.

[23] Paula J, Farah A (2019). Caffeine consumption through coffee: content in the beverage, metabolism, health benefits and risks. Beverages.

[24] Watson EJ, Coates AM, Kohler M, Banks S (2016). Caffeine consumption and sleep quality in Australian adults. Nutrients.

[25] Rochat C, Eap CB, Bochud M, Chatelan A (2020). Caffeine consumption in Switzerland: results from the first national nutrition survey menu CH. Nutrients.

[26] Frary CD, Johnson RK, Wang MQ (2005). Food sources and intakes of caffeine in the diets of persons in the United States. J Am Diet Assoc.

[27] Roehrs T, Roth T (2008). Caffeine: sleep and daytime sleepiness. Sleep Med Rev.

[28] Goldstein A, Kaizer S, Whitby O (1969). Psychotropic effects of caffeine in man. IV. Quantitative and qualitative differences associated with habituation to coffee. Clin Pharmacol Ther.

[29] Yang A, Palmer AA, Wit H (2010). Genetics of caffeine consumption and responses to caffeine. Psychopharmacology (Berl).

[30] Davidson MG (2011). Herbal-caffeinated chewing gum, but not bubble gum, improves aspects of memory. Appetite.

[31] Paton C, Costa V, Guglielmo L (2015). Effects of caffeine chewing gum on race performance and physiology in male and female cyclists. J Sports Sci.

[32] Smith A (2009). Effects of caffeine in chewing gum on mood and attention. Hum Psychopharmacol.

[33] Wickham KA, Spriet LL (2018). Administration of caffeine in alternate forms. Sports Med.

[34] Sharma A, Volkmar FR (2013). Encyclopedia of Autism Spectrum Disorders.

[35] Aslani A, Jalilian F (2013). Design, formulation and evaluation of caffeine chewing gum. Adv Biomed Res.

[36] Uddin LQ (2021). Cognitive and behavioural flexibility: neural mechanisms and clinical considerations. Nat Rev Neurosci.

[37] Sahakian BJ, Morris RG, Evenden JL, Heald A, Levy R, Philpot M (1988). A comparative study of visuospatial memory and learning in Alzheimer-type dementia and Parkinson’s disease. Brain.

[38] Chin JM, Merves ML, Goldberger BA, Sampson-Cone A, Cone EJ (2008). Caffeine content of brewed teas. J Anal Toxicol.

[39] Rezaee E, Mirlohi M, Hassanzadeh A, Fallah A (2016). Factors affecting tea consumption pattern in an urban society in Isfahan, Iran. J Educ Health Promotion.

[40] Ebrahimzadeh G, Nodehi RN, Alimohammadi M, Kahkah MR, Mahvi AH (2021). Monitoring of caffeine concentration in infused tea, human urine, domestic wastewater and different water resources in southeast of Irancaffeine an alternative indicator for contamination of human origin. J Environ Manage.

[41] Balentine DA, Harbowy ME, Graham HN, Balentine DA, Harbowy ME, Graham HN (2019). Caffeine.

[42] Darani HS, Ziarati P, Taherkhani M, Mousavi Z (2020). Method validation and determination of caffeine in drinks in the Iranian market by the HPLC. Med Sci.

[43] Javaheripour N, Shahdipour N, Noori K, Zarei M, Camilleri JA, Laird AR (2019). Functional brain alterations in acute sleep deprivation: An activation likelihood estimation meta-analysis. Sleep Med Rev.

[44] Medic G, Wille M, Hemels ME (2017). Short-and long-term health consequences of sleep disruption. Nat Sci Sleep.

[45] Cohen-Zion M, Shabi A, Levy S, Glasner L, Wiener A (2016). Effects of partial sleep deprivation on information processing speed in adolescence. J Int Neuropsychol Soc.

[46] Killgore WDS (2010). Effects of sleep deprivation on cognition. Prog Brain Res.

[47] Santhi N, Horowitz TS, Duffy JF, Czeisler CA (2007). Acute sleep deprivation and circadian misalignment associated with transition onto the first night of work impairs visual selective attention. PloS One.

[48] Taheri M, Arabameri E (2012). The effect of sleep deprivation on choice reaction time and anaerobic power of college student athletes. Asian J Sports Med.

[49] Hansen DA, Ramakrishnan S, Satterfield BC, Wesensten NJ, Layton ME, Reifman J (2019). Randomized, double-blind, placebo-controlled, crossover study of the effects of repeated-dose caffeine on neurobehavioral performance during 48 h of total sleep deprivation. Psychopharmacology (Berl).

[50] Spaeth AM, Goel N, Dinges DF (2014). The cumulative neurobehavioral and physiological effects of chronic caffeine intake: individual differences and implications for the use of caffeinated energy products. Nutr Rev.

[51] Souissi M, Chikh N, Affès H, Sahnoun Z (2018). Caffeine reversal of sleep deprivation effects on alertness, mood and repeated sprint performances in physical education students. Biol Rhythm Res.

[52] Souissi M, Chtourou H, Abedelmalek S, Ghozlane IB, Sahnoun Z (2014). The effects of caffeine ingestion on the reaction time and short-term maximal performance after 36h of sleep deprivation. Physiol Behav.

